# Finished Whole-Genome Sequences of *Clostridium butyricum* Toxin Subtype E4 and *Clostridium baratii* Toxin Subtype F7 Strains

**DOI:** 10.1128/genomeA.00375-17

**Published:** 2017-07-20

**Authors:** Jessica L. Halpin, Karen Hill, Shannon L. Johnson, David Carlton Bruce, T. Brian Shirey, Janet K. Dykes, Carolina Lúquez

**Affiliations:** a Centers for Disease Control and Prevention, Atlanta, Georgia, USA; b Los Alamos National Laboratory, Los Alamos, New Mexico, USA

## Abstract

*Clostridium butyricum* and *Clostridium baratii* species have been known to produce botulinum toxin types E and F, respectively, which can cause botulism, a rare but serious neuroparalytic disease. Here, we present finished genome sequences for two of these clinically relevant strains.

## GENOME ANNOUNCEMENT

Some rare strains of *Clostridium butyricum* and *Clostridium baratii* carry the botulinum neurotoxin gene cluster (1–8). Botulinum toxin causes a rare but serious disease that produces a descending flaccid paralysis that can result in death if not treated (9).

*C. baratii* type F strain CDC51267 and *C. butyricum* type E strain CDC51208 were sequenced by Illumina and PacBio. A hybrid assembly method was utilized, and the sequences from both isolates were assembled using Velvet 1.2.08, HGAP 3, and Phrap 4.24. The coverages for *C. baratii* isolate CDC51267 were 357× by PacBio and 342× by Illumina. The coverages for *C. butyricum* isolate CDC51208 were 52.6× by PacBio and 319× by Illumina.

*C. baratii* serotype F strain CDC51267 was isolated from a food specimen associated with a foodborne botulism outbreak. The finished genome includes one plasmid, pNPD11_1 (accession no. CP014203), of 120,667 bp, with 25.3% G+C content and 119 coding regions. The chromosome (accession no. CP014204) size is 3,091,050 bp, with 28.4% GC content and 2,885 identified coding regions. The toxin gene was determined to be subtype F7 and was found within the plasmid.

*C. butyricum* serotype E strain CDC51208 was isolated from an infant botulism case. The resulting finished genome includes two plasmids of 9,567 bp (pNPD4_1, accession no. CP013240) and 820,516 bp (pNPD4_2, accession no. CP13238). These plasmids consist of 26.8% and 28.2% G+C content, as well as 8 and 763 coding sequences (CDSs), respectively. The chromosome (accession no. CP013239) has 3,809,831 bp, 28.8% G+C content, and 3,334 identified coding regions. The toxin gene was determined to be subtype E4 and was found within the pNPD4_2 plasmid.

### Accession number(s).

These finished sequences have been deposited in GenBank, with accession numbers CP014204 and CP014203 for *C. baratii* strain CDC51267, and accession numbers CP013238, CP013239, and CP013240 for *C. butyricum* strain CDC51208.
