# Erratum for Halpin et al., “Finished Whole-Genome Sequences of *Clostridium butyricum* Toxin Subtype E4 and *Clostridium baratii* Toxin Subtype F7 Strains”

**DOI:** 10.1128/genomeA.01050-17

**Published:** 2017-09-21

**Authors:** Jessica L. Halpin, Karen Hill, Shannon L. Johnson, David Carlton Bruce, T. Brian Shirey, Janet K. Dykes, Carolina Lúquez

**Affiliations:** a Centers for Disease Control and Prevention, Atlanta, Georgia, USA; b Los Alamos National Laboratory, Los Alamos, New Mexico, USA

## ERRATUM

Volume 5, no. 29, e00375-17, 2017, https://doi.org/10.1128/genomeA.00375-17. Page 1; line 22: “was found within the pNPD4_2 plasmid” should read “was found within the chromosome.”

